# Proteome-wide forced interactions reveal a functional map of cell-cycle phospho-regulation in *S. cerevisiae*

**DOI:** 10.1080/19491034.2024.2420129

**Published:** 2024-12-01

**Authors:** Cinzia Klemm, Guðjón Ólafsson, Henry Richard Wood, Caitlin Mellor, Nicolae Radu Zabet, Peter Harold Thorpe

**Affiliations:** a School of Biological and Behavioural Sciences, Queen Mary University of London, London, UK; b Department of Bioengineering, Imperial College London, London, UK; c Department of Biochemistry and Molecular Biology, BioMedical Center, Faculty of Medicine, University of Iceland, Reykjavík, Iceland; d National Heart and Lung Institute, Imperial College London, London, UK; e Blizard Institute, Barts and The London School of Medicine and Dentistry, Queen Mary University of London, London, UK

**Keywords:** Cdc5, Cdc7, CDK, cell cycle, phosphatases, phospho-regulation, synthetic physical interactions

## Abstract

Dynamic protein phosphorylation and dephosphorylation play an essential role in cell cycle progression. Kinases and phosphatases are generally highly conserved across eukaryotes, underlining their importance for post-translational regulation of substrate proteins. In recent years, advances in phospho-proteomics have shed light on protein phosphorylation dynamics throughout the cell cycle, and ongoing progress in bioinformatics has significantly improved annotation of specific phosphorylation events to a given kinase. However, the functional impact of individual phosphorylation events on cell cycle progression is often unclear. To address this question, we used the Synthetic Physical Interactions (SPI) method, which enables the systematic recruitment of phospho-regulators to most yeast proteins. Using this method, we identified several putative novel targets involved in chromosome segregation and cytokinesis. The SPI method monitors cell growth and, therefore, serves as a tool to determine the impact of protein phosphorylation on cell cycle progression.

## Introduction

A common characteristic of all living cells is their ability to multiply, by replicating and segregating genetic information during the cell division cycle. In eukaryotes, cell cycle entry, progression, and exit are orchestrated by several signaling cascades which monitor cell size, stage, and composition, as well as environmental factors such as nutrient availability [1]. Successful chromosome segregation – the equal separation of sister chromatids to nascent daughter cells – is a crucial step during cell division. Errors in chromosome segregation can result in genomic instability and aneuploidy, an abnormal number of chromosomes. Aneuploidy is commonly observed in cancer cells [2] and, if it occurs during meiotic cell division, can lead to congenital disorders. Yeasts are one of the simplest eukaryotic cells, and their fast and easy cultivation and vast genetic toolset make them an ideal model organism to study general cellular processes. Unsurprisingly, much of our knowledge about cell cycle regulation is derived from previous research in budding and fission yeast [3,4].

Cell cycle progression and chromosome segregation are strictly regulated by multiple pathways to prevent errors. One important mechanism that drives mitotic progression is the posttranslational modification of proteins involved in chromosome segregation by phosphorylation and dephosphorylation. Cyclin-dependent kinases (CDKs) are the key drivers of the cell cycle in eukaryotic cells and are highly conserved from yeast to humans [5]. In the budding yeast *Saccharomyces cerevisiae*, a single catalytic subunit, Cdc28, is activated and recruited to its substrate proteins by one of the nine cell cycle stage-specific cyclin subunits. CDK phospho-regulation is essential for cell cycle progression and peaks during mitosis to ensure the transition from early metaphase to anaphase. In the late anaphase, mitotic phosphorylation events are reversed by the rise of phosphatase activity which, ultimately, promotes exit from mitosis. Apart from CDK phospho-regulation, there are several other kinases involved in cell cycle progression, including the Dbf4-dependent kinase (DDK) [6], and the Polo-like kinase Cdc5 [7]. Both kinases act in conjunction with CDK at different stages of the cell cycle [8]. DDK is active from early S to M-phase, and together with CDK regulates initiation of DNA replication and meiotic recombination, blocks over-replication, and supports chromosome segregation [6,9,10]. Cdc5 is important for post-metaphase events during mitosis, driving anaphase, and mitotic exit [11–13].

To allow dynamic regulation of the proteome, phosphorylation events are reversed by phosphatases throughout the cell cycle. Most importantly, mitotic exit is shaped by the release of the Cdc14 phosphatase from the nucleolus to reverse widespread CDK phosphorylation events and facilitate cell division, effectively resetting cells to G0/G1 state [14–16]. More distinct processes, such as checkpoint release or mitotic progression, are regulated by the protein phosphatases 1 (PP1) and 2A (PP2A) [17–21]. PP2A can be further subdivided into PP2A^Cdc55^ and PP2A^Rts1^ depending on the regulatory subunit, each of which recruits the catalytic domain to specific target proteins.

Large-scale phospho-proteomics studies have helped to identify thousands of substrate proteins of cell cycle kinases and phosphatases in recent years [18,21–25]. However, studying cell cycle phospho-regulators and their role at specific cellular targets is often difficult. Protein–protein interaction screens often do not detect the transient and cell cycle regulated interactions between cell cycle phospho-regulators and their substrates. Using classical genetics can be challenging, as both overexpression and deletion or downregulation of kinases and phosphatases often result in pleiotropic effects on multiple cellular processes. On the other hand, using phospho-mutants to mimic a specific phosphorylation state is useful to characterize effects on individual substrate proteins, but does not reflect the dynamic nature of phosphorylation events and could force the cell to adapt to the mutation. Forced recruitment of extra copies of phospho-regulators to a desired cellular target structure overcomes some of these limitations and can be used to identify specific phenotypes caused by the interaction of regulator and substrate protein. Previous experiments using this approach included forced CDK phosphorylation by fusing cyclin proteins to putative CDK substrates [26,27]. The Synthetic Physical Interactions (SPI) method applies the principle of forced protein recruitment in a high-throughput genome-wide scale in *S. cerevisiae* [28,29]. Using the SPI system, interactions are forced by a GFP nanobody, also known as GFP-binding protein (GBP), fused to gene of interest (GOI), which is then recruited to a GFP-tagged protein [30–32]. A set of potential phospho-regulators has been identified in genome-wide SPI screens with complexes involved in chromosome segregation, the kinetochore, and the spindle pole body [33,34]. Interestingly, only a small number of kinases and phosphatases produced SPI phenotypes when recruited to the kinetochore, and in some cases, regulatory rather than catalytically active subunits were identified in these screens. These were mostly conserved phospho-regulators known to be involved in cell cycle progression, many of which were shown to target the kinetochore. For Mps1, Cdc5, and Cdc14, the SPI method has proven to be useful to characterize their roles at the kinetochore in more detail [29,35,36]. These experiments showed that, overall, kinetochore subunits are affected differently by the forced recruitment of phospho-regulators. For example, Mps1 recruitment to subunits of the kinetochore KMN network produced growth defects in a SAC-dependent manner. Forced association of Cdc5 to the kinetochore COMA complex subunit Ame1 led to metaphase arrest and a de-clustered morphology of outer kinetochore subunits, and this phenotype has been linked to misregulation in centromeric transcription. Recruitment of Cdc5 to Mtw1 led to S/G2-phase arrest of cells when Cdc5-GBP expression was conditionally induced in asynchronous cells. In metaphase-arrested cells, forced association of Cdc5 and Mtw1 led to a reduced sister-kinetochore distance. A similar phenotype was observed in human cells when the human homolog of Cdc5, PLK1, was forcibly recruited to HEC1 (Ndc80 in budding yeast) [37]. Cdc14 recruitment to the MIND complex produced growth defects by preventing CDK-dependent phosphorylation of Dsn1 [33]. Thus, the SPI method is a powerful tool to unravel mechanisms of phospho-regulation important for cell cycle progression in vivo.

In this study, we have applied the SPI methodology to systematically recruit the cell cycle kinases CDK, DDK, and Cdc7 to most of the *S. cerevisiae* proteome. With this method, we were able to identify distinct interactions resulting in growth defects for kinase and phosphatase recruitment. Using SPI, we identified new putative targets of phospho-regulation and update the current model of cell cycle phospho-regulation.

## Materials and methods

### Yeast strains

Strains are listed in Table S1. The GFP library is a collection of 4159 strains derived from BY4741 (*MAT***a** *his3∆1 leu2∆0 met15∆0 ura3∆0*) [31], which is currently distributed by ThermoFisher Scientific. Each strain contains one open reading frame (ORF) with the DNA sequence of GFP inserted at the 3’-end, thus expressing a protein with a C-terminal GFP tag, and a downstream HIS3MX cassette as a selection marker. The universal donor strain (UDS, W8164–24, *MATα CEN1–16::Gal-KI-URA3 can1∆100 his3∆11,15 leu2∆3,112 LYS2 met17∆ trp1∆1 ura3∆1 RAD5*) was received from the Rothstein lab [38]. The *CEN1-16:Gal-KI-URA3* genotype contains a counter-selectable *URA3:GAL* promoter cassette derived from *Kluyveromyces lactis* at every centromere, which leads to chromosome destabilization on galactose media due to elevated transcription through centromeres. Furthermore, the addition of 5-fluoroorotic acid (5-FOA) leads to cell death of strains with the *URA3* gene as 5-FOA is converted to 5-fluorouracil. Plasmids for SPI analysis were transformed into the UDS and selected on synthetic media without leucine and glucose as the primary carbon source.

### Plasmids

Plasmids are listed in Table S2. All plasmids used for SPI analysis were derived from pWJ1512, a single-copy CEN plasmid that contains a *LEU2* gene as a selection marker and a *CUP1* promoter sequence [38]. The *CUP1* promoter is characterized by a low, constitutive activity that can be elevated. Plasmids were generated by gap-repair cloning or NEBuilder plasmid assembly combining the linearized donor plasmid with transforming PCR products. GOI plasmids were modified by site-directed mutagenesis to assess the role of protein activity in SPI phenotypes. For further characterization of SPI phenotypes, the pCUP1 promoter was exchanged with a repressible pMET3 promotor, which promotes protein expression only in absence of methionine [39]. All plasmids were validated before SPI screening by Sanger sequencing (GENEWIZ).

### Selective ploidy ablation for synthetic physical interactions

The impact of protein–protein interactions on yeast growth was analyzed on a proteome-wide scale using the Synthetic Physical Interactions (SPI) method [29]. Screens were performed with the ROTOR robot pinning platform (Singer Instruments), and yeast colonies were pinned in in 96-, 384-, or 1536-arrays onto rectangular Singer Plus Plates (Singer Instruments). The colony used in all SPI screens was 1536 with a pinning pressure of 25–30%, 2 pinning repeats, a pinning diameter of 0.3 mm for source plates and 0.1 mm for target plates with 2 rotation cycles. The pinning diameter was increased for lawn source plates to ensure the transfer of a sufficient number of cells.

**Figure 1.**
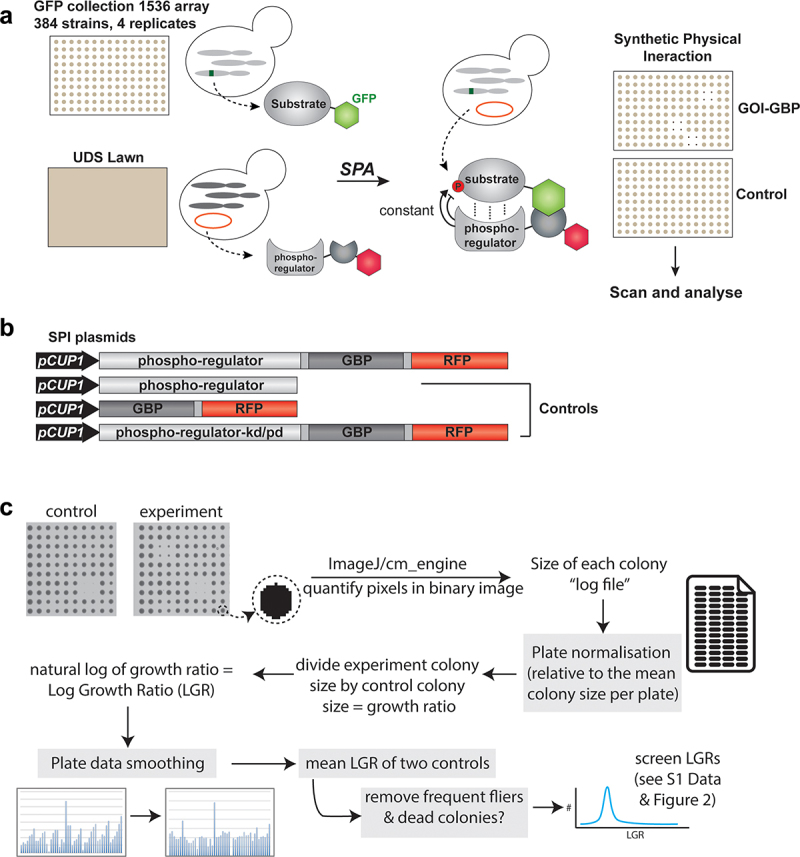
SPI screening of cell cycle phospho-regulators. a) Schematic showing workflow of SPI screening using the SPA method. UDS and GFP-tagged strains are mated and UDS chromosome counter selected using SPA. This results in haploid GFP-tagged cells containing SPI plasmids. Growth phenotypes are evaluated by colony growth on agar plates. b) Map of phospho-regulator and control plasmids used in this study. c) Schematic showing the computational and mathematical workflow used to analyze the scanned plates to derive Log Growth Ratios for each GFP strain. The steps inside gray boxes are optional in the ScreenGarden software or analysis.

Selective ploidy ablation (SPA) was developed by the Rothstein lab as a method for high-throughput plasmid transfer in budding yeast [38] and adapted for SPI analysis. Colonies were grown at 1536 density overnight at 30°C. ‘Small-scale’ *MAD3* and *BFA1* deletion-specific SPI screens, were performed in replicates of 16, genome-wide screens with 4 replicates per strain. The *MAT***a** GFP collection was then mated with a lawn of the *MATα* UDS strain containing SPI plasmids on YPD plates for a minimum of 8 h. The diploid colonies were then copied onto rectangular SC GAL -leucine plates and grown for 24 h and subsequently pinned onto SC GAL -leucine plates containing 4.3 mM 5-FOA to revert diploid cells back to haploids. After 48–72 h of incubation at 30°C, images of plates were taken using an Epson V750 Pro desktop flatbed scanner (Seiko Epson Corporation). The readout of SPI screens is colony growth compared to expression of the gene of interest (GOI control) and recruitment of GBP on its own (GBP control) (Figure 1b). Protein–protein interactions which cause a growth defect relative to both controls are defined as SPIs. Pixel values of the plate image were quantified using the CMengine of *ScreenMill* [40] and plates were compared and statistically analyzed using the ScreenGarden shinyR application [41].

### Bioinformatic analysis

Protein localization and GO slims of SPIs were defined using data from the Saccharomyces Genome Database (SGD) and YeastMine [42,43]. SPIs were visualized in the global yeast genetic interaction network, to identify relations between affected proteins. Calculation of Spearman’s Rank correlation and principal component analysis (PCA) compared to previous SPI screens [34,36,44] was performed using RStudio. The Cluster 3.0 software was used for centroid-linkage clustering of SPIs [45] and data was visualized using TreeView 1.1.6 [46]. Gene ontology (GO) enrichments were performed using GOrilla [47] using a target and background list approach. Enrichments are identified with a hypergeometric overrepresentation test with a p-value cutoff of 10–3. Enrichment terms with broad descriptions or great similarities were excluded.

### Fluorescence microscopy

Cells were grown to logarithmic (log) phase overnight in a shaking incubator at 23°C in appropriate growth media. In the case of using yeast strains with a conditional promoter system, log-phase cultures were pelleted and washed by centrifugation at 4000 rpm and RT for 5 min. Pellets were then resuspended in media containing the specific compound to induce gene expression and grown for further 2–4 h. Cells were then pelleted and washed to promote mitotic progression of synchronized cells. 3 µl of cells were then pipetted onto a microscopy slide and embedded in 0.7% agarose (low melting point) dissolved in growth media to ensure a depth of 6–8 µm between slide and coverslip. Single images were taken with a Zeiss Axio imager Z2 microscope (Carl Zeiss AG) using a 63 × 1.4 NA oil immersion lens and illuminated with a Zeiss Colibri LED illumination system (CFP = 445 nm, GFP = 470 nm, YFP = 505 nm, RFP = 545 nm). Differential interference contrast (DIC) prisms were used to enhance the bright field contrast and the light was captured using a Hamamatsu Flash 4.0 Lte CMOS camera with FL-400 (6.5 µm pixels, binned 2 × 2). Exposure times were adjusted to ensure that signal intensities remained below saturation. All images have a resulting pixel size of 206 nm in x and y, a z step size of 300 nm and an effective dynamic range of 30,000 gray levels. Images shown in the figures were processed using FIJI/ImageJ the Icy BioImage Analysis unit (version 2.0.3.0) which also uses the ImageJ software [48,49].

## Results

### Synthetic physical interactions identify crucial targets of cell-cycle phospho-regulation

**Figure 2.**
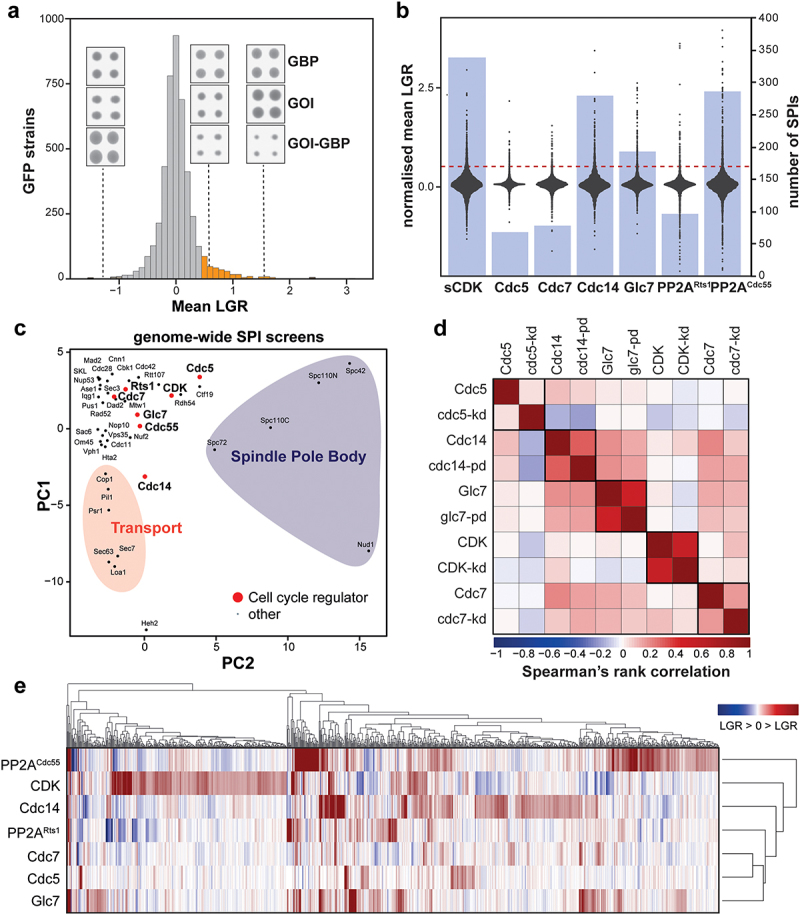
Phospho-regulators produce unique sets of SPIs. a) Plot showing mean LGR distributions resulting from a typical SPI screen. Data are mostly distributed around zero, with a positive tail indicating growth defects. The orange bars indicate LGRs >0.4. The insets show example images of colonies that result in the indicated LGRs, GOI, and GBP are controls, whereas GOI-GBP is the experiment (see Figure 1b). b) Results of phospho-regulator SPI screens, with the violin plots showing the data distribution (left axis) and the blue bars showing the number of SPIs per screen (right axis). c) PCA analysis comparing screens performed in this study to previous SPI screens. d) Spearman’s Rank correlation shows a correlation between active and inactive versions of each phospho-regulator. Darker red shading indicates a stronger positive correlation, blue shading shows a negative correlation. e) Hierarchical clustering of proteins that are annotated as SPIs in at least one screen (917 proteins) shows relatively low overlap (29%) of SPIs between different phospho-regulators. The Cluster 3.0 software was used for centroid-linkage clustering of SPIs [45], and data was visualized using TreeView 1.1.6 [46].

To get a more comprehensive understanding of phospho-regulation events during the budding yeast cell cycle, we extended our set of kinase and phosphatase SPI screens to include mitotic CDK (a fusion protein of the strong activating cycling Clb2 and the catalytic subunit Cdc28), Cdc7 (the active domain of DDK), and the phosphatases Cdc14, PP1 (the active subunit Glc7) and the regulatory subunits of PP2A, Cdc55, and Rts1. We created plasmids expressing these phospho-regulators. We also included data derived from a previous SPI screen with the Polo-like kinase Cdc5 in our data analysis [33]. In brief, the SPI system utilizes the mating-based Selective Ploidy Ablation (SPA) [38] method, in which a plasmid is transferred from a universal donor strain (UDS) to an array of yeast colonies with different genotypes, in this case, a collection of more than 4000 yeast strains with an open reading frame (ORF) fused to GFP (Figure 1a). After mating, diploid stains can express the SPI plasmids, encoding a phospho-regulator that is fused to both red fluorescent protein (RFP) and a GFP binding protein (GBP) [30]. This phospho-regulator -GBP-RFP fusion protein, will bind to the GFP-tagged proteins with high affinity. Diploids can be reverted to haploid yeast with the GFP yeast collection as genetic background, by destabilizing the UDS genome, which contains a *GAL* promoter and *URA3* marker adjacent to each centromere. Selecting on galactose and the addition of 5-fluorotic acid leads to ablation of the UDS genome. Finally, haploid strains with GBP-GFP interactions are tested for their ability to form colonies on solid agar, to detect inhibition of cell cycle progression. SPI analysis is performed using the active phospho-regulator -GBP-RFP fusion, the phospho-regulator alone (GOI control), GBP-RFP alone (GBP control) and an inactive version of the kinase/phosphatase fused to GBP-RFP, of which the latter three serve as controls (Figure 1b). Except for Cdc55 and Rts1, the regulatory domains of PP2A. To ensure successful kinase and phosphatase recruitment using the SPI system, we analyzed RFP-GFP colocalization using a set of cellular markers for different subcellular compartments (Figure S1). We then performed SPI screens with 1536 colonies per plate and 4 replicates per strain, totaling to 12 plates per plasmid, which are screened for small colonies indicating growth defects (Figure S2). Pixel values representing colony sizes were quantified using *ScreenMill’s* CM Engine [40] and plate-correction, normalization, and statistical analysis were performed using the ScreenGarden [41] software (Figure 1c). This produced density curves of log growth ratios (LGRs) of each screen, with most colonies showing no difference in growth between the recruited phospho-regulator and controls (Figure 2a,b). LGR’s of 0.4 indicate a 50% decrease in colony size, as described by Olafsson and Thorpe [28], which we consider as an empirical threshold for strains negatively affected by phospho-regulator recruitment, henceforth referred to as ‘SPIs’. Compared to GBP and GOI controls, we detected 338 SPIs for CDK (Clb2-Cdc28), 77 SPIs for DDK (Cdc7) and 68 SPIs for the Polo-like kinase Cdc5. Screening with Cdc14 produced 279 SPIs, PP1 (Glc7) resulted in 192 SPIs, and PP2A produced 284 and 95 SPIs with its regulatory Cdc55 and Rts1 subunits, respectively (Data S1). Eighty-seven percent (789 out of 915) of protein interactions that produce SPI phenotypes are annotated phospho-proteins from phospho-proteomics data (Data S1). Although physical interactions are a poor predictor of kinase/phosphatase substrates, we found that 11% (107 out of 915) of SPIs had a physical interaction with one or more phospho-regulators (Data S1).

### Phospho-regulator recruitment produces distinct SPI phenotypes but affect the same cellular processes and components

To characterize kinase and phosphatase SPI screens, we first compared our results with previously conducted SPI screens using Principal Component Analysis (PCA) (Data S2). These former screens include protein recruitment to different subcellular targets, generated by Berry and colleagues to obtain a cellular map of forced protein interactions [44], and screens with proteins of the kinetochore and the spindle pole body, which are a part of the mitotic spindle [29,34,36]. We also included screens with the kinase Cbk1, involved in cytokinesis, the cell-wall polarity GTPase Cdc42 and DNA repair protein Rtt107. PCA analysis revealed that most phospho-regulators cluster with proteins of the kinetochore, a nuclear complex, and other proteins involved in cell cycle processes (Figure 2c). Notably, the phosphatase Cdc14 is closer to a cluster of proteins involved in cellular transport, including Cop1, Sec67, and Sec7. Next, we wanted to see whether most SPIs are dependent upon phospho-regulator activity, by comparing screens of active and inactive kinases and phosphatases using Spearman’s rank correlation (Figure 2d). Interestingly, we found that screens correlated most closely with their inactive counterparts for all regulators tested, most strikingly for CDK and Glc7. We assume that the recruitment of an inactive regulator might prevent binding of the active endogenous protein, hence preventing phospho-regulation completely. Therefore, proteins sensitive to both, lack of phosphorylation, and constitutive phosphorylation can be affected by kinase- or phosphatase-dead protein recruitment. On the other hand, regulators that are active as dimers such as Cdc14, could lead to activation via recruitment of an endogenous, active version. Despite this possibility, we were able to show in a previous study that constitutive recruitment of inactive Cdc14 leads to increased phosphorylation of the MIND complex of the yeast kinetochore [29].

To compare the SPIs resulting from the phospho-regulator screens, we performed hierarchical clustering of all proteins that produced a SPI with one or more kinases and phosphatases (Figure 2e, Data S3). Strikingly, this resulted in distinct clusters for phospho-regulators, especially CDK, Cdc5, and Glc7. The general overlap of SPIs was also relatively low of around 29% (267 of 915). This indicates that distinct protein phosphorylation events are important for cell cycle progression and growth. These data show that SPIs with different phosphatases only partially overlap. Notably, recruiting the two different regulatory subunits of PP2A resulted in very different SPIs even though both, Cdc55 and Rts1, recruit the same catalytic domains (Pph1 and Pph2).

**Figure 3.**
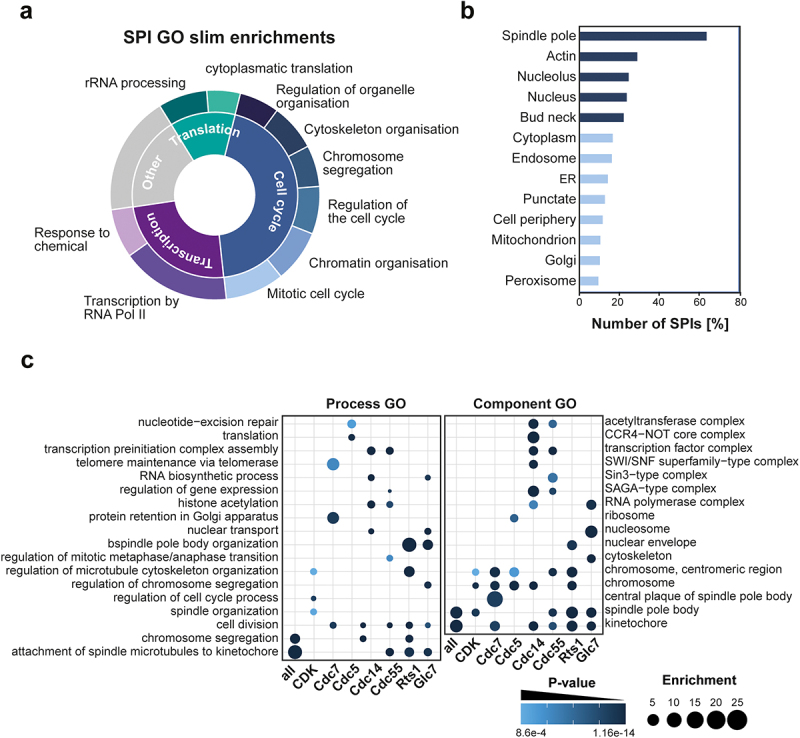
SPIs are enriched for similar processes and components. a) The sunburst chart summarizes GO slim term analysis. GO slims were enriched for processes involving the cell cycle, transcription, and translation (inner ring), which are specified further in the outer ring. b) Bar plot showing the analysis of cellular localization of SPI proteins. The dark bars indicate localizations that are increased in the SPIs compared to the ratio of the whole GFP collection. Light blue bars indicate decreased abundance. c) GO enrichment analysis for cellular processes and components, highlighting p-values and enrichment scores. GO enrichment was performed using the combined list of SPIs (‘all’) or for each regulator individually.

We then wanted to characterize the proteins affected by phospho-regulator recruitment. To do so, we performed Gene Ontology (GO) enrichment analysis using YeastMine [42] and extracted GO slim terms to obtain a general overview of the targets (Figure 3a, Data S4). We found that the majority of proteins (44%) affected by kinase and phosphatase recruitment were involved in the cell cycle, with mitotic cell cycle, chromatin organization, cell cycle regulation, chromosome segregation, cytoskeleton organization, and regulation of organelle organization being enriched. Twenty-four percent of the proteins were involved in transcription and transcriptional response to chemicals or translation and rRNA processing (12%). We further identified cellular localization of the SPIs using the data generated from genome-wide microscopy screens of the yeast GFP collection [31]. We found a higher abundance of bud neck, nuclear, nucleolar, cytoskeletal, and spindle pole proteins in our SPI data than expected based on their ratio within the total GFP collection (Figure 3b, Data S5). To delve deeper into the processes and components affected by phospho-regulator recruitment, we performed GO enrichment using GOrilla [47] (Figure 3c, Data S6). Despite the low overlap in SPIs between screens, all kinases and phosphatases tested produced SPIs enriched for proteins involved in chromosome segregation, spindle organization, and cell division, with the kinetochore, spindle pole bodies, and chromosomal proteins being generally enriched in most screens. Cdc14 and Cdc55 were additionally enriched for protein complexes involved in transcription, such as the SAGA-type complex and SWI/SNF complex. Cdc5 SPIs were enriched for translation and DNA repair processes.

### Human orthologs of phospho-regulator SPIs include genes involved in human disease

**Figure 4.**
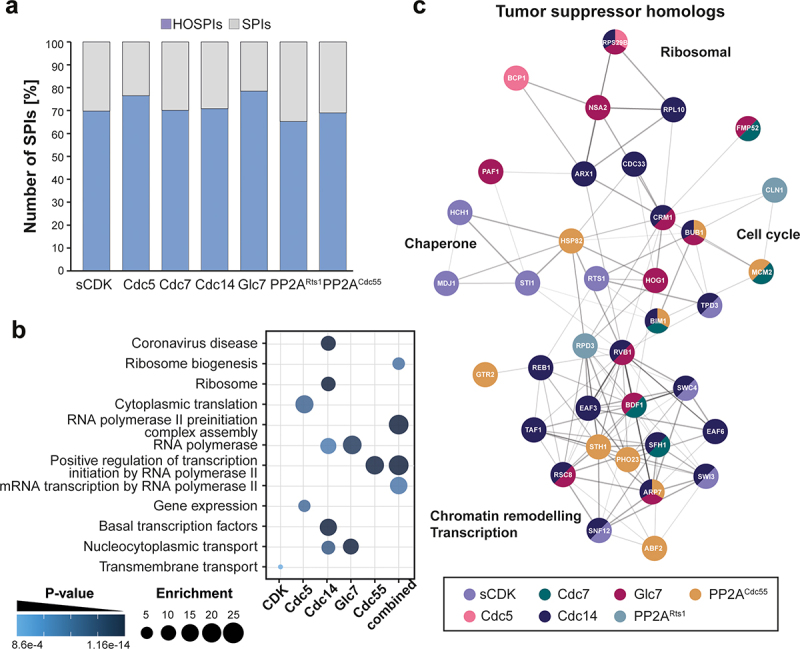
HOSPI analysis identifies potential targets involved in human disease. a) The bar chart shows the percentage of SPI proteins that have human orthologs for each regulator screen. b) GO enrichment of human orthologs reveals enrichment for proteins involved in transcription and transcriptional regulation. The plot highlights p-values and enrichment scores. c) Network showing HOSPIs with tumor suppressor genes. Coloring of the nodes accounts for SPIs observed in our screens. Edges highlight relationships between different genes. The network was generated using the STRING database [51] and prepared using Cytoscape [52].

*S. cerevisiae* is a simple model organism for eukaryotic cells and many proteins and regulatory mechanisms are conserved in higher organisms, including humans. All kinases and phosphatases investigated in this study are highly conserved and play important roles for cell cycle progression and disease in human cells. We have previously shown that SPI screening can be advantageous to study molecular players in human disease, by recruiting selected SARS-CoV-2 proteins to the budding yeast proteome [50]. Therefore, we wanted to see whether we can identify conserved putative targets of cell cycle regulation that can lead to human disease. We first quantified the number of conserved proteins within our SPIs. We found that approximately 70% of SPIs were conserved in human cells on average based on YeastMine (Figure 4a, Data S7). We termed these proteins Human Ortholog SPIs (HOSPIs). We then performed GO enrichment with the HOSPIs and found enrichments for transcription and transcriptional regulation, translation, ribosomal proteins, and transmembrane and nuclear transport (Figure 4b). Interestingly, we also found that a number of HOSPIs were known tumor suppressor genes (Figure 4c, Figure S3), which are known to interact with each other based on network analysis using the STRING database [51,53]. If phosphorylation or dephosphorylation activates these proteins constitutively, they could potentially prevent cellular growth in *S. cerevisiae* as they prevent uncontrolled growth in humans. This would also indicate that their functionality is dependent on cell cycle phospho-regulation by the respective regulator(s).

### SPI analysis reveals new aspects of cell cycle phospho-regulation

**Figure 5.**
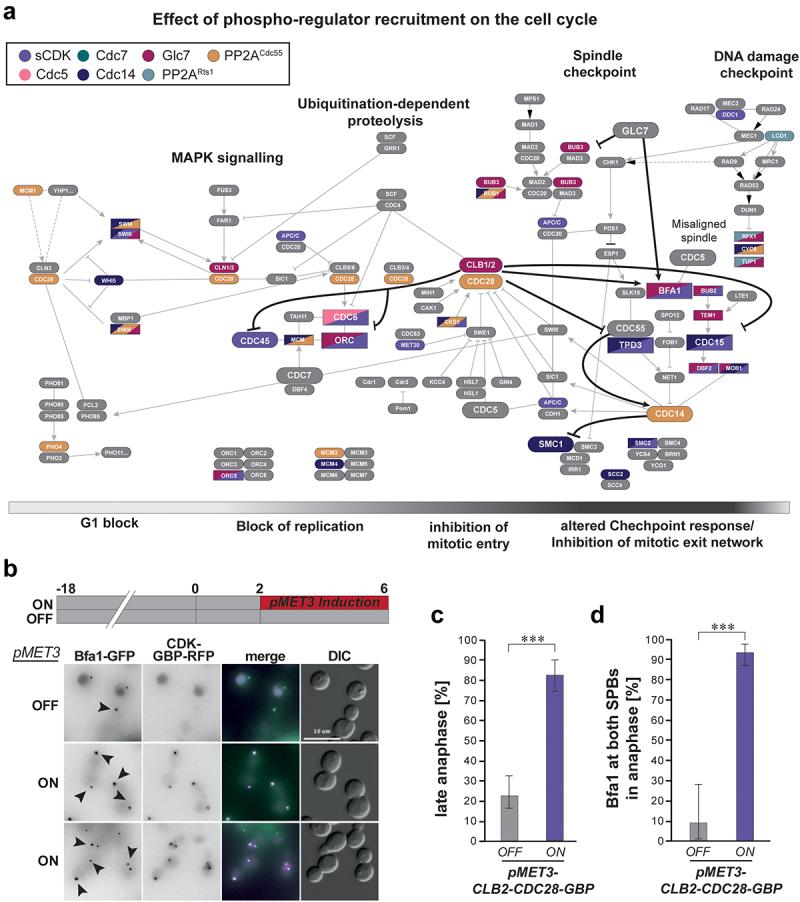
Phospho-regulator SPI screens identify new aspects of cell cycle regulation. a) KEGG network of *S. cerevisiae* cell cycle regulation showing novel aspects of cell cycle regulation suggested by SPI screening, highlighted by bold and black edges. Coloring of nodes represents SPI phenotypes with respective regulators identified in our screens. b) Micrographs showing the effect of CDK recruitment to Bfa1-GFP. When CDK is recruited to Bfa1, cells arrest in late anaphase and fail to divide. Bfa1 is also localized at both SPBs, rather than daughter cell SPBs only. c) Quantification of cells in the late anaphase. Error bars represent 95% confidence intervals and p-values were calculated using Fisher’s Exact test (*** = p-value <0.001). d) Quantification of Bfa1-GFP localization at both SPBs. Error bars represent 95% confidence intervals and p-values were calculated using Fisher’s Exact test (*** = p-value <0.001).

As shown in Figure 3c, proteins involved in cell cycle regulation are enriched in our SPI data. To see whether our data reveals potentially novel aspects of cell cycle regulation, we mapped SPIs onto the current model for cell cycle regulation in *S. cerevisiae* extracted from the KEGG database [54] (Figure 5a). We found that many known interactions between kinases and phosphatases were represented in our SPI data. Furthermore, we identified new potential aspects of cell cycle regulation (edges highlighted in bold, black). For example, the DNA replication initiation factor Cdc45 was sensitive to CDK recruitment in our screens, together with its interactors Cdc6 and Mcm2. Whilst phospho-regulation of Cdc6 has been shown to prevent reinitiation of replication and regulates Cdc6 degradation [55,56], direct regulation of Cdc45 has not been shown yet, although CDK activity is required for Cdc45 function [57]. Recent sensitive phospho-proteomics have detected a putative CDK site within the C-terminal region of Cdc45 [25], and our results suggest a direct regulation by CDK via this site. In accordance with a recent study, we also found that Cdc14 and Cdc55 recruitment to Mcm2 inhibited growth, demonstrating its importance for regulating replication via Cdc45-Mcm2-Cdc6 [58]. Interestingly, we also saw growth defects when Cdc5 was recruited to Cdc6. A clear regulation of this complex by Cdc5 has not been revealed yet, making this phenotype an interesting target for future research.

One role of the Glc7 phosphatase is to switch off the spindle assembly checkpoint (SAC), which ensures that chromosomes are only segregated when mitotic spindles are correctly attached to kinetochores [59]. In yeast, the SAC is dispensable indicating that the SPI phenotype observed when Glc7 is recruited to Bub3 is unlikely merely due to SAC malfunction. This indicates a potentially different regulatory mechanism of Bub3 which requires further investigation.

Our results also indicate Cdc14-dependent regulation of cohesin, as recruiting the phosphatase to Smc1 leads to growth defects. Cohesin is crucial for chromosome condensation, and cohesin cleavage is necessary for chromosome segregation [35,60]. Cleavage is mediated by the separase Esp1 in a CDK-dependent manner [61], which also triggers early Cdc14 release via the FEAR (14 early anaphase release) network [62]. Further investigation of the SPI phenotype is necessary to identify Cdc14’s direct effect on the cohesin complex.

Phospho-regulator recruitment also revealed new aspects of mitotic exit regulation. The MEN is a signaling cascade activated at the end of mitosis to promote the full release of Cdc14 from the nucleolus into the nucleus and cytoplasm [63,64]. The MEN is localized at SPBs, where Nud1 serves as a scaffold for the signaling cascade. The MEN is only active in the bud compartment, as the presence of the kinase Kin4 in mother cells activates the Bub1-Bfa1 complex, which suppresses MEN activation. Kin4 is excluded from the bud due to the presence of the GDP/GTP exchange factor Lte1, which promotes MEN activity [65,66]. At daughter SPBs (dSPBs), Bfa1 is recruited by Lte1 which leads to Bfa1 phosphorylation and inactivation by Cdc5 [13]. This phosphorylation is reversed by PP2A-Cdc55 [67]. Kin4 prevents Bfa1 accumulation at mother SPBs (mSPBs), which promotes Bub1-Bfa1 activity in the mother cell and silences MEN signaling at mSPBs. The GTPase Tem1 accumulates at dSPBs in the late anaphase to recruit and activate the kinase Cdc15. Cdc15 then phosphorylates the Mob1 Dbf2 kinase complex to promote its re-localization to the nucleus, where it mediates full Cdc14 release into the cytoplasm to drive mitotic exit and cell division [68]. CDK is known to phosphorylate and inhibit Cdc15 and Dbf2 to prevent premature exit from mitosis in metaphase when CDK concentration is at its peak [69,70]. Both forms of MEN regulation, the spatial regulation promoted by Lte1 and the temporal regulation by cyclin-CDK expression, are important to prevent early activation of the MEN pathway and, consequentially, premature exit from mitosis. We have recently published a spatiotemporal model of the MEN and shown the MEN is inhibited upon CDK recruitment only in combination with Bub2-Bfa1 activity [71], and CDK SPIs with most MEN components can be rescued by deleting BFA1 (Figure S4, Data S8).

Howell and colleagues’ spatial logical model also predicted that CDK activity prevents mitotic exit specifically when recruited to the dSPB, where MEN signaling is activated by Bub2-Bfa1 inhibition. Bub2-Bfa1 is inhibited by phosphorylation of Bfa1 by Cdc5 when Bub2-Bfa1 is localized at dSPBs. In the mother cell compartment, Bfa1 is phosphorylated by Kin4 in anaphase, which promotes the binding of Bmh1 (14-3-3) and subsequent dissociation of Bub2-Bfa1-Bmh1 from SPBs and protects Bfa1 from Cdc5 phosphorylation (Figure S4). Hence, CDK recruitment to Bub2-Bfa1 would specifically target MEN components at dSPBs, leading to failure of mitotic exit based on the spatial logical model. Indeed, CDK SPIs produced phenotypes when recruited to both subunits of the Bub2-Bfa1 complex (Figure S4). Furthermore, growth was not affected when CDK was recruited to the mother compartment-specific Kin4, supporting the hypothesis that CDK activity prevents MEN activity at dSPBs. To further investigate the phenotype of CDK recruitment to Bfa1, we transformed pMET3 inducible versions of CDK-GBP and GBP into Bfa1-GFP strains. Cells were highly sensitive to CDK recruitment when pMET3 expression was induced in low methionine concentration (Figure 5b). Fluorescence microscopy revealed that most Bfa1-GFP cells expressing CDK were arrested in late anaphase, suggesting a failure to progress through mitotic exit (Figure 5b,c and Data S9). Some cells also contained two Bfa1-GFP foci in the mother compartment, a phenotype which indicates SPOC activity [72,73]. Interestingly, despite differences in signal intensities, most cells expressing Clb2-Cdc28-GBP contained Bfa1 SPB foci in both mother and bud compartments (Figure 5b,d and Data S9). These data suggest that CDK recruitment to Bfa1 affects the MEN signaling pathway and prevents asymmetric localization of Bfa1 during anaphase.

**Figure 6.**
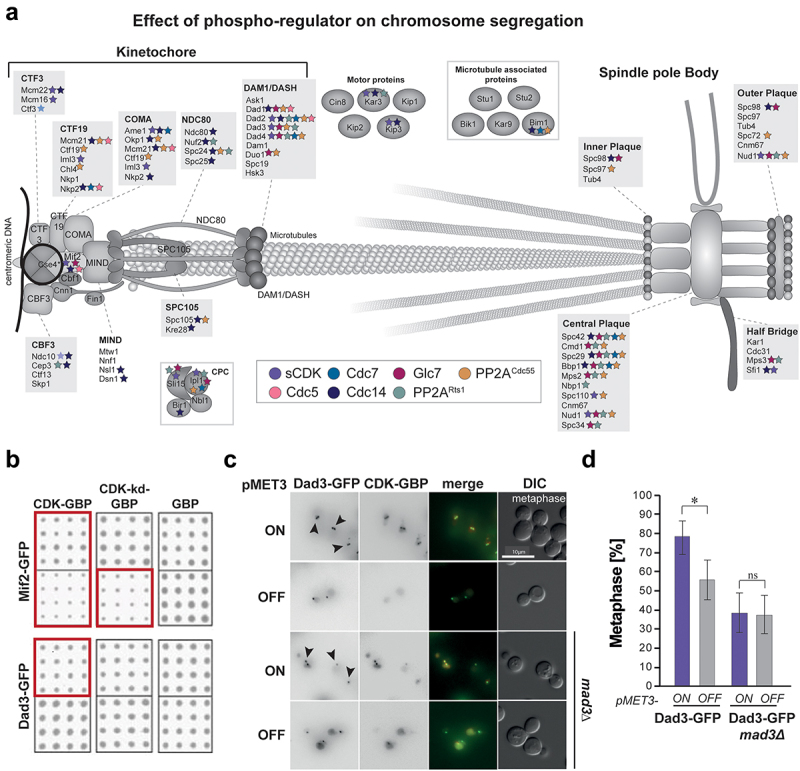
SPI identifies the kinetochore and spindle pole as important phospho-regulation targets for mitotic progression. a) Schematic of the kinetochore, microtubule, and spindle pole body highlighting phospho-regulator SPIs for each subunit in respective colors. b) Scans of selected SPIs from *MAD3* deletion screens. Growth defects are highlighted in red boxes. Mif2-GFP is affected more severely in SAC-deficient yeast, whereas the Dad3 SPI phenotype is rescued by *MAD3* deletion. c) Micrographs looking at Dad3-GFP CDK SPI phenotypes in wildtype and *mad3Δ* backgrounds using the inducible MET3 promoter SPI system. In the wildtype, cells arrest in metaphase upon CDK recruitment and hindering SAC activity rescues this phenotype. d) Quantification of microscopy data, showing the percentage of cells in metaphase. Error bars represent 95% confidence intervals and p-values were calculated using Fisher’s Exact test (* = p-value <0.05).

We mapped SPI phenotypes produced by phospho-regulator recruitment to highlight protein complexes involved in chromosome segregation (Figure 6a). Recruitment of CDK affected components of the constitutive centromere associated network (CCAN) at the kinetochore (Mif2, Ame1, Mcm22, and Iml3) and the DAM1/DASH complex (Dad2, Dad3, and Dad4). Furthermore, CDK recruitment led to growth defects with the motor protein Kar3, the chromosomal passenger complex (CPC) protein Sli15, the spindle pole body subunits Spc110, Nud1, and Sfi1. Forced association of Cdc14 affected components of the same complexes at the kinetochore as CDK, however, different subunits of the COMA complex (Okp1, Mcm21, and Nkp2) and the DAM1/DASH complex (Dad1 and Dad2) were affected by Cdc14 recruitment. In addition, cells were sensitive to Cdc14 recruitment to the MIND complex (Nsl1 and Dsn1) and, strikingly, all subunits of the microtubule-interacting complexes SPC105 (Spc105 and Kre28) and NDC80 (Ndc80, Nuf2 Spc24, and Spc25). Furthermore, Cdc14 produced overlapping SPIs to Clb2 Cdc28 with the motor proteins Kip3 and Kar3, in addition to the microtubule-associated protein Bim1. At SPBs, Cdc14 produced growth defects when recruited to Spc98 the central plaque proteins Spc42, Spc29, and Bbp1, and Sfi1 of the half-bridge. The nuclear pore complex component Nup192 was also sensitive to Cdc14 recruitment. Forced association of Glc7 produced SPI phenotypes with the DAM1/DASH complex (Dad2, Dad3, and Dad4) and Mif2 at the kinetochore. In addition, Glc7 produced SPIs with multiple SPB proteins (Spc98, Nud1, Spc42, Spc29, Bbp1, Mps2, and Spc34), components of the nuclear pore complex (Ndc1 and Nup192) and the CPC components Sli15 and Ipl1. Rts1 SPIs included the inner kinetochore subunits Ctf3, Cep3 and outer kinetochore subunits Nuf2 and Spc24 of the NDC80 complex and Dad2, Dad3, and Dad4 of the DAM1/DASH complex. Like CDK and Cdc14, Rts1 recruitment affected growth in Kar3-GFP cells. At SPBs Nud1, Spc29, Bbp1 Mps2, Nbp1, and Spc34 were sensitive to Rts1 recruitment and the nuclear pore protein Ndc1 produced a SPI phenotype. Furthermore, the CPC subunits Sli15 and Ipl1 were affected by Rts1 recruitment. Notably, Cdc55 recruitment produced more SPIs with kinetochore and SPB proteins compared to Rts1. Both regulatory subunits of PP2A are known to have distinct functions and the low correlation of our data suggests that their role is not exclusively linked to recruitment [18]. More widespread effects observed upon Cdc55 recruitment may indicate a higher affinity of Cdc55 to the catalytic subunit or more extensive regulation of Rts1-Pph21/22 interaction. Cdc5 and Cdc7 recruitment produced distinct SPIs with the Ctf19, COMA, and DAM1/DASH complexes, highlighting their crosstalk at the yeast centromere. Previous studies have also confirmed their involvement in centromeric regulation [74,75]. Cdc7 also produces SPIs at the SPBs, an interesting observation as Cdc7 is involved in the separation initiation network (SIN) in *Schizosaccharomyces pombe* at SPBs [76]. Contrarily, in *S. cerevisiae* Cdc5, but not Cdc7, has been attributed to regulation of mitotic exit at SPBs.

If the forced association of phospho-regulators affect phosphorylation sites involved in checkpoint activation, growth defects could be a consequence of the spindle assembly checkpoint (SAC). The SAC identifies incorrect kinetochore-microtubule attachments and promotes cell cycle arrest in metaphase by Mps1-mediated phosphorylation of KMN components [59]. Once microtubule attachments are formed properly, these proteins are dephosphorylated by Glc7 to promote cell cycle progression. In budding yeast, the SAC is a non-essential pathway and is only required for growth under conditions that inhibit spindle attachments. To address the impact of the SAC on SPI phenotypes, we abolished SAC signaling by performing SPI screens with cells lacking the MCC component Mad3 and compared these to colonies with an active SAC (Figure 6b and Data S10). If growth phenotypes are a consequence of SAC activation, these growth defects would be rescued in a *mad3Δ* background. Conversely, SAC-deficient yeast cells are more sensitive to weakened kinetochore-microtubule attachments, thus milder SPI phenotypes can be amplified if forced association of phospho-regulators affects kinetochore integrity and formation of microtubule attachments. Mif2-GFP was more sensitive to CDK recruitment in *mad3Δ* background and affected upon recruitment of a catalytically inactive version of CDK, indicating SAC-independent cell cycle regulation. We have extensively characterized the Mif2-CDK phenotype and shown that Mif2 phosphorylation by CDK stabilizes Mif2 at kinetochores, indicating that Mif2 phospho-regulation is important for protein turnover [77]. Conversely, the Dad3 SPI phenotype was rescued by SAC inhibition. A known target of CDK at the DAM1/DASH complex is Ask1, and Ask1 phosphorylation has been shown to promote the stability of kinetochore-microtubule attachments [78]. Hence, CDK activity is thought to counteract the Ipl1 error correction pathway which targets the Dam1 subunit. In human cells, CDK1 phosphorylates the functionally conserved Ska complex, which leads to Ska recruitment to kinetochores and promotes mitotic progression [79]. To characterize the effect of CDK recruitment to the DAM1/DASH complex, we transformed the conditional pMET-CDK-GBP plasmid into Dad3-GFP strains in cells with and without the SAC component Mad3. Fluorescent imaging showed that cells expressing CDK were indeed enriched in metaphase and this phenotype was reduced upon SAC depletion (Figure 6c,d and Data S11), indicating that CDK recruitment to the DAM1/DASH complex could indeed promote SAC activation.

## Discussion

### Forced association of phospho-regulators affects components involved in transcription, chromosome segregation, and mitotic exit

Using the SPI method, we have identified many target proteins sensitive to kinase and phosphatase recruitment (summarized in Data S12). In some cases, these SPIs may not result from physiological effects, since the SPI method forces an interaction. However, many SPIs were proteins involved in processes that are regulated by CDK and the phosphatases Cdc14, PP1, and PP2A. For example, all phospho-regulators screened in this study produced growth defects when recruited to different subunits of the kinetochore. Second, SPBs, also known as microtubule organizing centers (MTOCs) or centrosomes in mammals, were affected by regulator recruitment. SPBs contain many phospho-proteins and SPB phosphorylation by CDK and other cell cycle kinases such as Mps1 and Cdc5 plays an essential role in SPB duplication and assembly [80–83]. Previous studies have also shown that cell cycle-dependent phosphorylation of γ-tubulin and γ-tubulin receptor subunits regulates spindle length and is essential for cell cycle progression [84,85]. Both, Cdc14 and Glc7 recruitment to the γ-tubulin Spc98 led to growth deficiency and CDK recruitment to the γ-tubulin receptor Spc110 produced a SPI phenotype, which may provide a means to manipulate microtubule nucleation. Besides their role in microtubule organization, SPBs are important signaling hubs. For example, double-strand break (DSB)-mediated release of Cdc14 promotes dephosphorylation of Spc110 to stabilize metaphase spindles and promote DSB-SPB tethering important for DNA repair [86]. Although these studies underline the impact of SPB phospho-regulation to drive cell cycle progression, to date only a few of the hundreds of detected phospho-sites of SPB components have been characterized [83]. Phospho-regulator recruitment produced one or more phenotypes with most SPB components and could serve as a reference point for future experiments to unravel the role of dynamic SPB phosphorylation throughout the cell cycle. Additionally, SPI analysis revealed further potential functions of phospho-regulators. For example, Glc7 recruitment to proteins involved in cell polarity caused growth defects, and Glc7 is known to promote cell integrity, polarization, bud morphology, and cytokinesis [87,88]. Cdc14 recruitment affected proteins involved in transcription. Notably, the recruitment of Cdc14 to RNA-polymerase II subunits produced only weak SPI phenotypes. Whilst this is surprising, it highlights the difference between forced protein recruitment, which remains subject to other regulatory mechanisms, such as dimerization in the case of Cdc14, compared to phospho-mutant analysis. However, several RNA-polymerase III subunits were affected by Cdc14 recruitment (namely Rpc17, Rpc53, Rpc82, and Rpc37). Although Cdc14 activity peaks at the mitotic exit, Cdc14 is part of the regulator of nucleolar silencing and telophase exit (RENT) complex which mediates transcriptional silencing. As a part of RENT, Cdc14 promotes telomere segregation by repressing RNA-polymerase II-mediated transcription [89,90]. The growth defects caused by the recruitment of Cdc14 to other proteins involved in transcription identified in the SPI screen are less well characterized. However, proteomic analysis has revealed that Cdc14 is a part of the mediator complex interactome [91]. The strong impact of Cdc14 on transcriptional complexes observed with the SPI method suggests a more profound role of Cdc14 in transcriptional regulation. Hence, further research to characterize the nature of these putative interactions could reveal new functions of Cdc14 apart from promoting mitotic exit in budding yeast.

### Recruitment of phosphatase subunits produced unique SPI phenotypes

Traditionally, phosphatase activity has been described as promiscuous compared to kinase activity, as kinases outnumber phosphatase proteins extensively. However, research from the last decades has proven that phosphatase activity is far more complex [92]. For example, PP1 and PP2A activity is regulated by binding to different regulatory and structural subunits that modulate phosphatase activity in a spatiotemporal manner and Cdc14 is confined to the nucleolus until mitotic exit. Consistent with the notion that PP2A^Rts1^ and PP2A^Cdc55^ are functionally different, our SPI screens with PP2A’s regulatory subunits Cdc55 and Rts1 demonstrated quite different groups of SPIs, even though the catalytic subunit is shared between them. Overall, SPI recruitment of phosphatase subunits to the yeast proteome revealed specific sensitivity of GFP-tagged proteins with different phosphatases, despite affecting related processes (Figure 2b). This observation supports the notion that phosphatases act on a defined set of substrates throughout the cell cycle. Consistently, in vitro analysis of Cdc14 activity revealed a strong substrate preference for phosphorylated SPx(K/R) motifs, which is also the full consensus sequence of CDK, and Cdc14 mediated dephosphorylation of CDK sites is shaped by the catalytic efficiency of specific substrates and a PxL motif, which facilitates Cdc14 recruitment [93–95]. Research in mammalian cells has shown that the counterpart of PP2A^Cdc55^, PP2A^B55^, displays substrate specificity for phospho-threonine flanked by a bipartite polybasic recognition sequence, whereas PP2A^B56^, the human homologue of PP2A^Rts1^, recognizes a LxxIxE [96–98]. Despite these new findings of phosphatase specificity, phospho-proteomic analysis of mitotic exit in budding yeast reported close cooperation of Cdc14, PP2A^Rts1^, and PP2A^Cdc55^ [18]. This study found that many proteins and phospho-sites are dephosphorylated by more than one phosphatase, making it challenging to identify the impact of specific phosphatases on mitotic exit. As SPI screens monitored the effect of phosphatase recruitment rather than the dephosphorylation status of proteins, the SPI data derived from genome-wide studies indicate specific roles of mitotic phosphatases crucial for cell growth. Hence, despite their largely overlapping target sites, the unique phenotypes observed in these SPI screens suggest that phosphatases play important roles in promoting distinct aspects of mitotic exit.

Taken together, we show that SPI screens are a powerful method to identify cellular phospho-regulation targets important for cell cycle progression and growth. In combination with advances in phospho-proteomics, our tool is a powerful method to elucidate functional aspects of cell cycle regulation. Furthermore, SPI allows the phenotypic characterization in living cells and using an inducible recruitment system facilitates tracking of cellular responses to phospho-regulator recruitment.

### Analysis of conserved SPIs (HOSPIs) could be used to study human disease

HOSPI analysis identified several conserved human proteins that play a role in cancer and other diseases. Future research could focus on these targets and see if phospho-site mutations are commonly observed in cancer cells in these proteins. Moreover, since the read-out of SPI screening is growth, the SPI methodology could be performed using human proteins, by replacing GOI and GFP-tagged proteins with conserved counterparts in yeast. Equally, recent research has adapted a methodology similar to SPI for mammalian cells to investigate disease phenotypes directly in higher organisms [99]. Although this study focused on protein degradation, the methodology could be easily adapted to study phospho-regulation in higher eukaryotes. Overall, using SPI to study protein phosphorylation is a powerful tool with possible applications beyond molecular yeast research.

## Supplementary Material

Supplementary Tables and Figures .docx

## Data Availability

The data generated during the study is available at figShare DOI: 10.6084/m9.figshare.27135972

## Declaration of publication

Much of the work described in this manuscript has been published in a thesis submitted in fulfillment of a PhD degree by Cinzia Klemm at Queen Mary University of London.
