# Supplementary material for: Proteome-wide forced interactions reveal a functional map of cell-cycle phospho-regulation in S. cerevisiae
Source: Nucleus. 2024 Dec 1;15(1):2420129. doi: 10.1080/19491034.2024.2420129 (PMC11622623; doi:10.1080/19491034.2024.2420129)
Supplement: Supplementary Tables and Figures .docx [file KNCL_A_2420129_SM6800.docx]

**Supplementary Tables and Figures**

**Table S1: Yeast strains generated and used in this study.**

| Strain | Genotype | Source |
| --- | --- | --- |
| W8164-2B | *MATα CEN1-16::Gal-KI-URA3* | Reid et al. 2011^1^ |
| GFP library | *MAT***a** *his3Δ1 leu2Δ0 met15Δ0 ura3Δ0 XXX-GFP:HIS3* | Huh et al. 2003^2^ |
| BY4741 | *MAT***a** *his3Δ1 leu2Δ0 met15Δ0 ura3Δ0* | Bachmann et al. 1998^3^ |
| BY4742 | *MATα his3Δ1 leu2Δ0 met15Δ0 ura3Δ0* | Bachmann et al. 1998^3^ |
| K3080 | *MAT***a** *ura3Δ1 his3Δ11,15 leu2Δ3 112 trp1Δ1, ade2 can1Δ100 Gal+, Δclb1, Δclb3::TRP1, Δclb4::HIS3, clb2-VI* | Amon et al. 1993^4^ |
| T414 | *MAT***a** *NUF2-GFP::HIS3 mad3Δ::KAN* | Olafsson and Thorpe 2015^5^ |
| T415 | *MAT***a** *NNF1-GFP::HIS3 mad3Δ::KAN* | Olafsson and Thorpe 2015^5^ |
| T416 | *MAT***a** *MTW1-GFP::HIS3 mad3Δ::KAN* | Olafsson and Thorpe 2015^5^ |
| T417 | *MAT***a** *CDC14-GFP::HIS3 mad3Δ::KAN* | Olafsson and Thorpe 2015^5^ |
| T418 | *MAT***a** *KRE28-GFP::HIS3 mad3Δ::KAN* | Olafsson and Thorpe 2015^5^ |
| T419 | *MAT***a** *NSL1-GFP::HIS3 mad3Δ::KAN* | Olafsson and Thorpe 2015^5^ |
| T420 | *MAT***a** *CEP3-GFP::HIS3 mad3Δ::KAN* | Olafsson and Thorpe 2015^5^ |
| T421  T422 | *MAT***a** *DAD3-GFP::HIS3 mad3Δ::KAN*  *MAT***a** *BIK1-GFP::HIS3 mad3Δ::KAN* | Olafsson and Thorpe 2015^5^ Olafsson and Thorpe 2015^5^ |
| T423 | *MAT***a** *BUB3-GFP::HIS3 mad3Δ::KAN* | Olafsson and Thorpe 2015^5^ |
| T424 | *MAT***a** *MIF2-GFP::HIS3 mad3Δ::KAN* | Olafsson and Thorpe 2015^5^ |
| T425 | *MAT***a** *CBF1-GFP::HIS3 mad3Δ::KAN* | Olafsson and Thorpe 2015^5^ |
| T426 | *MAT***a** *SLI15-GFP::HIS3 mad3Δ::KAN* | Olafsson and Thorpe 2015^5^ |
| T427 | *MAT***a** *AME1-GFP::HIS3 mad3Δ::KAN* | Olafsson and Thorpe 2015^5^ |
| T428 | *MAT***a** *MCM21-GFP::HIS3 mad3Δ::KAN* | Olafsson and Thorpe 2015^5^ |
| T429 | *MAT***a** *CTF19-GFP::HIS3 mad3Δ::KAN* | Olafsson and Thorpe 2015^5^ |
| T430 | *MAT***a** *STU2-GFP::HIS3 mad3Δ::KAN* | Olafsson and Thorpe 2015^5^ |
| T431 | *MAT***a** *DSN1-GFP::HIS3 mad3Δ::KAN* | Olafsson and Thorpe 2015^5^ |
| T541 | *MAT***a** *CEP3-GFP::HIS3 mad3Δ::KAN* | Olafsson and Thorpe 2015^5^ |
| T542 | *MAT***a** *KRE28-GFP::HIS3 mad1Δ::KAN* | Olafsson and Thorpe 2015^5^ |
| T543 | *MAT***a** *NDC80-GFP::HIS3 mad1Δ::KAN* | Olafsson and Thorpe 2015^5^ |
| T544 | *MAT***a** *AME1-GFP::HIS3 mad1Δ::KAN* | Olafsson and Thorpe 2015^5^ |
| T545 | *MAT***a** *BUB1-GFP::HIS3 mad1Δ::KAN* | Olafsson and Thorpe 2015^5^ |
| T546 | *MAT***a** *SPC105-GFP::HIS3 mad1Δ::KAN* | Olafsson and Thorpe 2015^5^ |
| T548 | *MAT***a** *his3Δ1 leu2Δ0 met15Δ0 ura3Δ0 CSE4-GFPint::HIS3MX6* | Lisa Berry^6^ |
| T594 | *MAT***a** *BUB1-GFP::HIS3 mad3Δ::KAN* | Olafsson and Thorpe 2015^5^ |
| T595 | *MAT***a** *SPC105-GFP::HIS3 mad3Δ::KAN* | Olafsson and Thorpe 2015^5^ |
| T596 | *MAT***a** *NDC80-GFP::HIS3 mad3Δ::KAN* | Olafsson and Thorpe 2015^5^ |
| T603 | *MAT***a** *DAD1-GFP::HIS3 mad3Δ::KAN* | Olafsson and Thorpe 2015^5^ |
| T604 | *MAT***a** *DAD4-GFP::HIS3 mad3Δ::KAN* | Olafsson and Thorpe 2015^5^ |
| T605 | *MAT***a** *BIM1-GFP::HIS3 mad3Δ::KAN* | Olafsson and Thorpe 2015^5^ |
| T766 | *MAT***a** *DAD2-GFP::HIS3 mad3Δ::KAN* | This study |
| T776 | *MAT***a** *KAR3-GFP::HIS3 mad3Δ::KAN* | This study |
| T777 | *MAT***a** *DBF2-GFP::HIS3 bfa1Δ::KAN* | This study |
| T778 | *MAT***a** *NUD1-GFP::HIS3 bfa1Δ::KAN* | This study |
| T732 | *MAT***a** *CDC15-GFP::HIS3 bfa1Δ::KAN* | Howell et al. 2020^7^ |
| T733 | *MAT***a** *MOB1-GFP::HIS3 bfa1Δ::KAN* | Howell et al. 2020^7^ |
| T779 | *MAT***a** *TEM1-GFP::HIS3 bfa1Δ::KAN* | Howell et al. 2020^7^ |
|  |  |  |

**Table S2: Plasmids generated and used in this study.**

| ^89^Genotype (all contain ARS209, CEN6 and AMP) | Source |
| --- | --- |
| *pCUP1 LEU2* | Reid et al. 2011^1^ |
| *pCUP1 GBP-RFP LEU2* | Olafsson and Thorpe 2015^5^ |
| *pGAL1 LEU2* | Olafsson and Thorpe 2015^5^ |
| *pGAL1 GBP-RFP LEU2* | Olafsson and Thorpe 2015^5^ |
| *pCUP1 CDC14 LEU2* | Olafsson and Thorpe 2015^5^ |
| *pCUP1 CDC14-GBP-RFPLEU2* | Olafsson and Thorpe 2015^5^ |
| *pCUP1 CDC14-CS-GBP-RFP LEU2* | Olafsson and Thorpe 2015^5^ |
| *pCUP1 CDC28 LEU2* | This study |
| *pCUP1 CDC28-GBP-RFP LEU2* | This study |
| *pCUP1 GLC7 LEU2* | This study |
| *pCUP1 GLC7-GBP LEU2* | This study |
| *pCUP1 RTS1 LEU2* | This study |
| *pCUP1 RTS1-GBP-RFP LEU2* | This study |
| *pCUP1 CDC28-K40L-GBP-RFP LEU2* | This study |
| *pCUP1 GLC7-L74P-GBP-RFP LEU2* | This study |
| *pCUP1 RTS1-D372R-S547A-W657A-GBP-RFP LEU2* | This study |
| *pCUP1 CLB2-CDC28 LEU2* | This study |
| *pCUP1 CLB2-CDC28-GBP-RFP LEU2* | This study |
| *pCUP1 CLB2-CDC28-K40L-GBP-RFP LEU2* | This study |
| *pCUP1 CDC5 LEU2* | Mishra et al., 2019^8^ |
| *pCUP1 CDC5-GBP LEU2* | Mishra et al., 2019^8^ |
| *pCUP1 CDC5-kd-GBP LEU2* | Mishra et al., 2019^8^ |
| *pCUP1 CDC7 LEU2* | Mishra et al., 2021^9^ |
| *pCUP1 CDC7-GBP LEU2* | Mishra et al., 2021^9^ |
| *pCUP1 CDC7-kd-GBP LEU2* | Mishra et al., 2021^9^ |
| *pMET3 GBP-RFP LEU2* | This study |
| *pMET3 GBP-RFP LEU15 LEU2* | This study |
| *pMET3 CLB2-GBP-GBP-RFP LEU2* | This study |
| *pMET3 CLB2-GBP-GBP-RFP LEU15 LEU2* | This study |

**Supplementary Figure Legends**

**Figure S1: Recruitment of phospho-regulators using Synthetic Physical Interactions**

A) Schematic of forced association of plasmid-expressed phospho-regulators tagged to GBP in GFP strains. If the phospho-regulator is recruited to a substrate protein, constitutive phosphorylation or dephosphorylation can be triggered.

B-F) Micrographs of GBP and phospho-regulators recruited to five different cellular localisations. The five GFP strains were chosen based on their well-established localisation within the cell and clear fluorescent signal. The green and red channels are shown in greyscale for clarity, the scale bars are 10 μM.

**Alt Text** Figure S1 shows micrographs of five different GFP strains (Hta1, Sec26, Tom70, Mtw1 and Spc42) with different GBP encoding plasmids.

**Figure S2: Example SPI screen plate with 384 strains and 1536 colony density.**

Example SPI screen plate derived from the CDK recruitment screen. Growth phenotypes are converted to pixel values compared to plates with GBP and CDK-only plasmids using the CMEngine^10^ and ScreenGarden^11^.

**Alt Text** Figure S2 Shows an example of raw growth data from our synthetic physical interaction screen.

**Figure S3: Network of tumour suppressor HOSPIs.**

HOSPIs contain several tumour suppressor genes, involved in transcription, cell cycle regulation and translation. HOSPIs were identified using YeastMine^12^. Shading and thickness of edges represent confidence of protein-protein interactions. Networks were generated using the STRING database^13^ and prepared using Cytoscape^14^.

**Alt Text** Figure S3 shows a graph illustrating protein-protein interactions between the human orthologs of tumour suppressor-related genes identified in our synthetic physical interaction screen.

**Figure S4: CDK recruitment to daughter, but not mother SPBs, results in growth defects.**

A) The schematic shows the regulation of the Bfa1-Bub2 complex during mitosis. The complex is phosphorylated by CDK and Kin4 for dynamic regulation of its localisation at mSPBs. Kin4 presence at mSPBs leads to Bfa1-Bub2 dissociation and thus MEN activation.

B) Crops of Bub1, Bfa1 and Kin4 SPI phenotypes when GBP or CDK is recruited.

**Alt Text** Figure S4 illustrates the regulation of Bfa1-Bub2 during mitosis and shows the growth effect when CDK is recruited to some of these proteins.

**Supplementary References**

1. Reid, R. J. D. *et al.* Selective ploidy ablation, a high-throughput plasmid transfer protocol, identifies new genes affecting topoisomerase I-induced DNA damage. *Genome research* **21**, 477–86 (2011).

2. Huh, W. K. *et al.* Global analysis of protein localization in budding yeast. *Nature* **425**, 686–691 (2003).

3. Brachmann, C. B. *et al.* Designer deletion strains derived from Saccharomyces cerevisiae S288C: a useful set of strains and plasmids for PCR-mediated gene disruption and other applications. *Yeast* **14**, 115–132 (1998).

4. Amon, A., Tyers, M., Futcher, B. & Nasmyth, K. Mechanisms that help the yeast cell cycle clock tick: G2 cyclins transcriptionally activate G2 cyclins and repress G1 cyclins. *Cell* **74**, 993–1007 (1993).

5. Ólafsson, G. & Thorpe, P. H. Synthetic physical interactions map kinetochore regulators and regions sensitive to constitutive Cdc14 localization. *Proceedings of the National Academy of Sciences of the United States of America* **112**, 10413–8 (2015).

6. Berry, L. K., Ólafsson, G., Ledesma-Fernández, E. & Thorpe, P. H. Synthetic protein interactions reveal a functional map of the cell. *eLife* **5**, e13053 (2016).

7. Howell, R. S. M., Klemm, C., Thorpe, P. H. & Csikász-Nagy, A. Unifying the mechanism of mitotic exit control in a spatiotemporal logical model. *PLOS Biology* **18**, e3000917 (2020).

8. Mishra, P. K. *et al.* Cell cycle-dependent association of polo kinase Cdc5 with CENP-A contributes to faithful chromosome segregation in budding yeast. *Molecular Biology of the Cell* **30**, 1020–1036 (2019).

9. Mishra, P. K. *et al.* Cdc7-mediated phosphorylation of Cse4 regulates high-fidelity chromosome segregation in budding yeast. *Molecular biology of the cell* **32**, ar15 (2021).

10. Dittmar, J. C., Reid, R. J. & Rothstein, R. *Open Access SOFTWARE ScreenMill: A Freely Available Software Suite for Growth Measurement, Analysis and Visualization of High-Throughput Screen Data*. *BMC Bioinformatics* vol. 11 353 http://www.biomedcentral.com/1471-2105/11/353 (2010).

11. Klemm, C., Howell, R. S. M. & Thorpe, P. H. ScreenGarden: a shinyR application for fast and easy analysis of plate-based high-throughput screens. *BMC Bioinformatics* **23**, 60 (2022).

12. Balakrishnan, R. *et al.* YeastMine--an integrated data warehouse for Saccharomyces cerevisiae data as a multipurpose tool-kit. *Database : the journal of biological databases and curation* **2012**, (2012).

13. Szklarczyk, D. *et al.* The STRING database in 2021: customizable protein-protein networks, and functional characterization of user-uploaded gene/measurement sets. *Nucleic acids research* **49**, D605–D612 (2021).

14. Shannon, P. *et al.* Cytoscape: a software environment for integrated models of biomolecular interaction networks. *Genome Res* **13**, 2498–2504 (2003).
